# Endometrial cancer: mapping the global landscape of research

**DOI:** 10.1186/s12967-020-02554-y

**Published:** 2020-10-12

**Authors:** Dörthe Brüggmann, Katja Ouassou, Doris Klingelhöfer, Michael K. Bohlmann, Jenny Jaque, David A. Groneberg

**Affiliations:** 1 Department of Gynecology and Obstetrics, Goethe-University, Theodor-Stern Kai 7, 60590 Frankfurt, Germany; 2 Division of Female Health and Preventive Medicine, Institute of Occupational Medicine, Social Medicine and Environmental Medicine, Goethe-University, Frankfurt, Germany; 3 Department of Obstetrics and Gynecology, Department of Obstetrics and Gynecology St, Elisabeth Hospital, Loerrach, Germany; 4 Department of Obstetrics and Gynecology, Keck School of Medicine of USC, Los Angeles, CA USA

**Keywords:** Endometrial carcinoma, Density equalizing mapping, Socio-economic analysis

## Abstract

**Background:**

From a global viewpoint, endometrial cancer belongs to the most common female cancers. Despite the heavy burden of diseases and numerous unanswered questions, no detailed pictures of the global structure of endometrial cancer research are available so far. Therefore, this malignancy was reviewed using the New Quality and Quantity Indices in Science (NewQIS) protocol.

**Methods:**

Using NewQIS, we identified endometrial carcinoma related research published in the Web of Science from 1900–2015 (P1) and from 2016–2020 (P2). Item analysis was performed with regard to research activity. Also, semi-qualitative aspects and socio-economic benchmarks were visualized using density equalizing mapping.

**Results:**

In total, 9,141 from 1900–2015 and 4,593 from 2016–2020 endometrial cancer related studies were identified with the USA having the largest numbers of publications, citations, institutions, as well as the highest country-specific h-Index concerning endometrial cancer research in both periods. In contrast to other fields of cancer research, the two East Asian countries Japan and China followed concerning total research activities until 2015. From 2016 until 2020, China was found in short distance to the USA and was ranked second. In the socio-economic analysis, European countries were in prominent positions. Greece published 579.83 endometrial carcinoma-related articles per billion US-$ GDP, Finland (527.29), Sweden (494.65), Israel (493.75), and Norway (367.85) followed in the ranking. Density equalizing mapping visualized that large parts of Africa, Asia and South America with a high burden of disease played almost no visible role in the endometrial cancer research activities.

**Conclusions:**

Endometrial cancer research activity is continuously increasing from a global viewpoint. However, the majority of original articles is published by authors based in high-income countries. Together with the finding that the research field of public health does only play a minimal role, our study points to the necessity that global health aspects should be introduced to endometrial cancer research.

## Background

As stated by the FIGO platform Global Library of Women’s Medicine, endometrial cancer is a common gynecologic malignancy affecting hundreds of thousands of women globally [1]. In 2018 more than 382,000 new cases were diagnosed, and nearly 90,000 women died worldwide from the disease (https://gco.iarc.fr/today/data/factsheets/cancers/24-Corpus-uteri-fact-sheet.pdf**)**. The numbers are increasing annually, with an disproportional growth in developed countries [1, 2]. Known risk factors are obesity-related exposure to estrogens, older age (≥ 55 years), tamoxifen use, early age at menarche and late-onset menopause, while diabetes is still debated [3–8]. When worldwide incidences are compared, the highest numbers are seen for Canada and the United States of America (USA) [2, 8]. In the USA, endometrial carcinoma is the fourth most common malignant tumor in women. It represents the most frequently diagnosed gynecologic cancer [1] and is linked to an extremely high individual and socio-economical disease burden.

Although mortality has decreased over the years, health care providers face challenges in managing this disease,  which originates from the endometrial lining of the uterine cavity [8]. For example, patient care became more complex since changes in histological classifications complicate the comparison of randomized trials, treatment decisions and the predictions of prognosis [8]. Also, many unanswered research questions remain ranging from treatment toxicity, diagnostic procedures to adjuvant therapy [8–10]. Nevertheless, significant scientific progress related to endometrial cancer has been made during the last decade, e.g. researchers defined new cancer subtypes according to the genomic characterization of the malignancy based on the cancer genome atlas [11]. But data on its true impact on public health and disease burden on a global scale are scarce. Also, no systematic evaluation of global research output was published so far.

Depiction the research productivity on a global level is essential not only for individual scientists but also policy makers to plan future research projects, prioritize research needs and resources as well as allocate funding in a transparent and fair way [12]. Hence, it was the aim of this study to investigate the global research productivity on endometrial cancer by this scientometric in-depth analysis based on the established New Quality and Quantity Indices in Science (NewQIS) computing platform [13]. Using this previously published and reliable approach, the objectives of the present study included (a) the analysis of the worldwide research activity by means of publication output, chronological and geographical developments on endometrial cancer since 1900, (b) the depiction of related scientific networks and the (c) the assessment of country-specific socio-economic figures and their relation to research strength.

## Methods

### NewQIS study platform

This assessment of global and country-specific endometrial cancer research patterns was conducted using the established and previously described NewQIS platform [13, 14] which was founded in 2007–2008 at the Humboldt-University´s school of medicine Charité. Over the past decade, over 80 peer-reviewed studies were conducted by the use of NewQIS in areas ranging e.g. from public health issues [15] to infectious diseases [16] and obstetrics and gynecology [17]. Hence, validated protocols allow the objective, precise and reliable analysis of the research output in an efficient and standardized way.

### Data source

As previously described, the Web of Science (WoS core collection, Clarivate Analytics) was used as data source for the analysis of endometrial cancer-specific publications. The main reason for choosing the WoS was the ability to use the Citation Report function [17].

### Search strategy

In order to specifically assess publications related to endometrial cancer and increase the validity of our search, a “**titl**e” search was performed. We identified endometrial cancer related publications for two time periods. 1900 to 2015 (named P1) and 2016 until 2020 (P2) was performed. The search term endometr* AND (neoplasm* or cancer* or carcinoma*) NOT endometriosis NOT endometrioma was used. As previously described, the filter option “document type” was used in order to restrict our search to “**original articles**” [13]. Thereby, research activity was aimed to be analyzed rather than the activity to publish news, reviews or other articles not related to original research studies on endometrial carcinoma. Duplicate publications were excluded using the MS Access duplication identification tool.

### Data analysis and categorization

As previously described, we analyzed the set of data according to the following criteria: country origin, citations, journals, cited references, authors, year published and subject categories. We also calculated Hirsch-Indices [17] for all countries (HI) and citation rates of all countries (CR, number of total country-specific citations per total endometrial carcinoma publication number). We employed a “modified” Hirsch-Index to assess the recognition of a single country’s research performance in the scientific community. This index was developed by Jorge Hirsch in 2005 as a proxy measure to gauge the quality of a single author’s work [18]. Further, we calculated the relative proportion of articles published by authors affiliated with the 10 most productive countries in endometrial cancer research and depicted their proportional change from 1981 to 2020.

### Socio-economic analysis

We compared research activity to socioeconomic features as previously described for other NewQIS studies [17]. Therefore, we analyzed research productivity in relation (a) to the countries’ gross domestic product (GDP) per capita, (b) to the countries’ total economic power index GDP per 1000 billion US-$ and (c) to the countries’ population sizes. The figures were retrieved from the *World Factbook* of 2014 [19]. We also calculated research activity in relation to estimated new cases of endometrial cancer per year. These data were retrieved from the GLOBOCAN 2018 project of the International Agency for Research on Cancer (IARC) of the World Health Organization (WHO) [20].

### Density equalizing map projections

We used density equalizing map projections (DEMP) to visualize the results. Here, the global distribution of investigated parameters is depicted by the generation of anamorphic world maps. For this purpose, we generated DEMPs based on the algorithms published by Gastner and Newman [21] after transfer of the metadata into a Microsoft® Access® database and parameter analysis. We utilized the “ArcGIS Cartogram” geoprocessing tool (https://www.arcgis.com) to build the anamorphic cartograms. The maps represent the territories of research publishing countries resized in proportion to the selected criteria (i.e. the number country-specific citations).

### Endometrial carcinoma international research networks

As discussed in previous studies, we also aimed to visualize an underlying international network of collaborations in endometrial cancer research. Therefore, all author affiliations were analyzed and chart diagrams were computed [17]. International articles were defined as “collaborative” when the affiliations of the publishing authors stated different countries. We generated a network graph, where the vectors represent the productivity of collaborations for pairs of countries. Also, we related the total count of collaborative items to the overall number of publications for each investigated country.

## Results

### General parameters

**Fig. 1:**
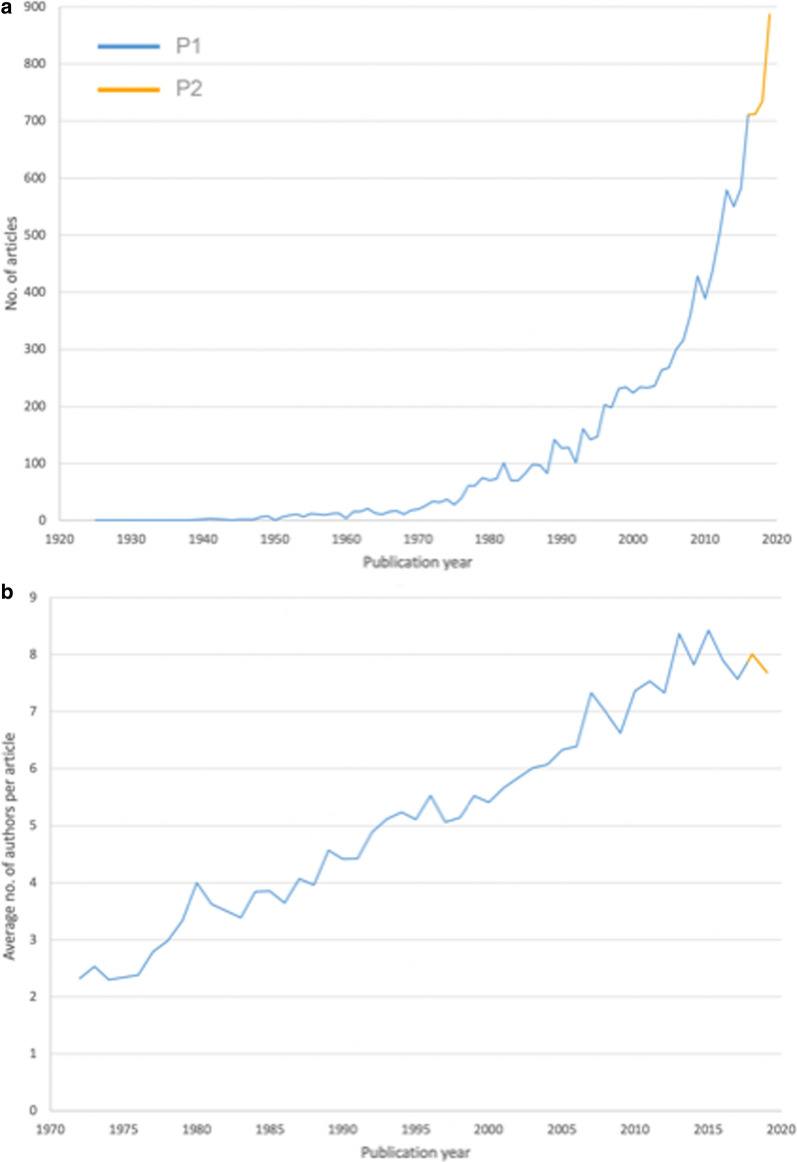
Chronological development. *P1* period from 1900–2015, *P2* period from 2016–2020. **a** Number of published items. **b** Average number of authors per publication

9,141 articles were published between 1900 and 2015. In P2, authors issued 4,593 articles on endometrial cancer in the WoS. Global publication activities increased slowly between 1900 and 1970 with maximal 21 articles per year. From 1970 onwards, the annual number increased more steeply and surpassed 100 annual articles for the first time in 1982 (n = 101). In 2015, 582 annual articles were identified. This number grew grew steadily and reached a peak with 886 articles published in 2019 (Fig. 1). The accelerated complexity of research is paralleled by the increasing number of scientists who are involved in each endometrial cancer study: Between 1972 and 2015, the average number of scientists who authored an article increased from 2.32 (1972) to 8.42 authors per study in 2015.

### Country-specific analysis

From 1900 until 2015, the USA published more than a third of the global research output related to endometrial carcinoma (36.4%, n = articles; n = 3,191). After 2015, the USA published 946 articles, which account for 20,7% of the total articles published between 2016 until 2020. Altogether, the USA was identified as the leading nation in this respective scientific field based on its remarkable productivity from 1900 until 2020. Until 2015, Japan accumulated 1,074 articles and occupied position 2, followed by China (n = 611). Together, these two Asian countries constituted 19.2% of all endometrial cancer-related publications. The most productive European country was Italy in forth position (n = 599), followed by Germany and Great Britain (n = 519 and n = 409). From 2016 until 2020, the ranking changed. China was found in short distance to the USA and was ranked second (n = 915), followed by Japan (n = 282) and Italy (n = 216). Turkey (n = 201) and Canada (n = 161) replaced the UK (n = 148, now position 7) and Germany (n = 142, position 8) in positions 5 and 6.

**Fig. 2:**
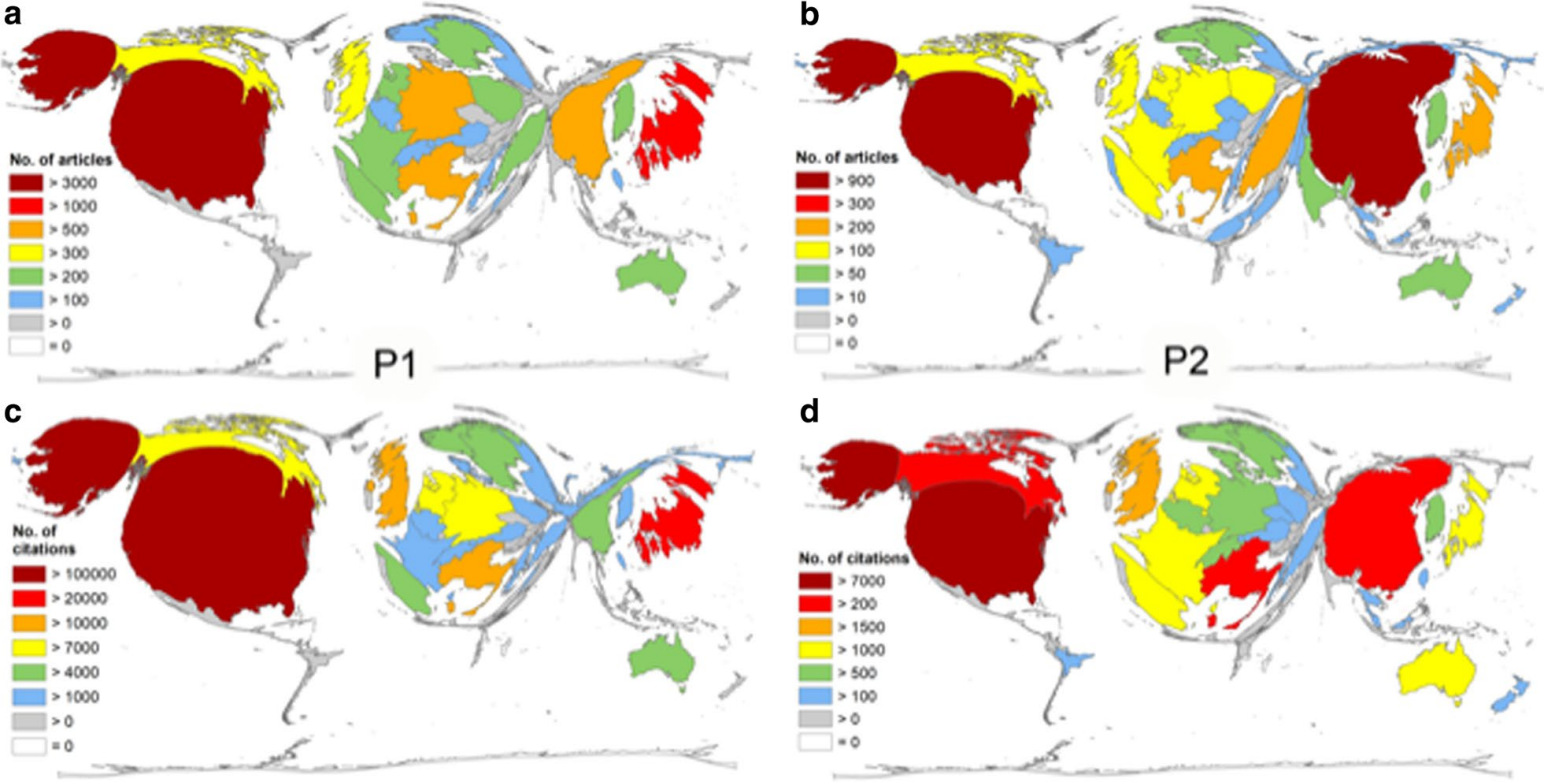
Density equalizing map projection of absolute numbers. Number of endometrial cancer publications per country in the period P1 = from 1900–2015 (**a**) and period P2 = period from 2016–2020 (**b**). Number of citations per country in the period P1 from 1900–2015 (**c**) and period P2 = period from 2016–2020 (**d**)

DEMP analyses of all published articles in the periods P1 and P2, led to a completely distorted world map. For P1, we found the main focus on North America with the USA, Asia with Japan and China and Western Europe with Italy, Germany and the UK (Fig. 2a). African countries occupy only minimal areas on the DEMP cartogram with Egypt and South Africa having n = 17 and n = 13 articles, respectively. For P2, the map (Fig. 2b) represents the identified increase in scientific productivity in China. Here, its territory grew remarkably in size.

### Citation analysis

Paralleling the global publication activity on endometrial cancer, the country-specific citation analysis showed a leading position of the USA in both evaluation periods. In P1, the articles by US-American authors were cited 103,737 times (44% of all citations, c = citations), 7,699 citations were identified for P2. Again, Japan was ranked second with 21,395 citations. Although China was the third most active country research activity, it was only the 10th most cited country (c = 5,476). Italy held the third position (c = 11,012), followed by the UK (c = 10,014) and Canada (c = 9,727). The Netherlands and Germany achieved 8,396 and 7,342 citations, respectively. Sweden was positioned at rank 8 (c = 6,770) and Norway followed (c = 5,783, Fig. 2c). The world map representing the country-specific number of citations changed compared to the research activity DEMP, specifically with Eastern Asia (China) being smaller. For P2, China improved its ranking to the second position (c = 4,620) followed by Canada (c = 2,336), Italy (c = 2,005), the UK (c = 1,804) and Japan (c = 1,406). The Scandinavian countries Norway (c = 778) and Sweden (c = 643) dropped to position 12 and 13 whereas the Netherlands and Germany and held positions 8 and 11 (formerly in positions 6 and 7, Fig. 2d).

**Fig. 3:**
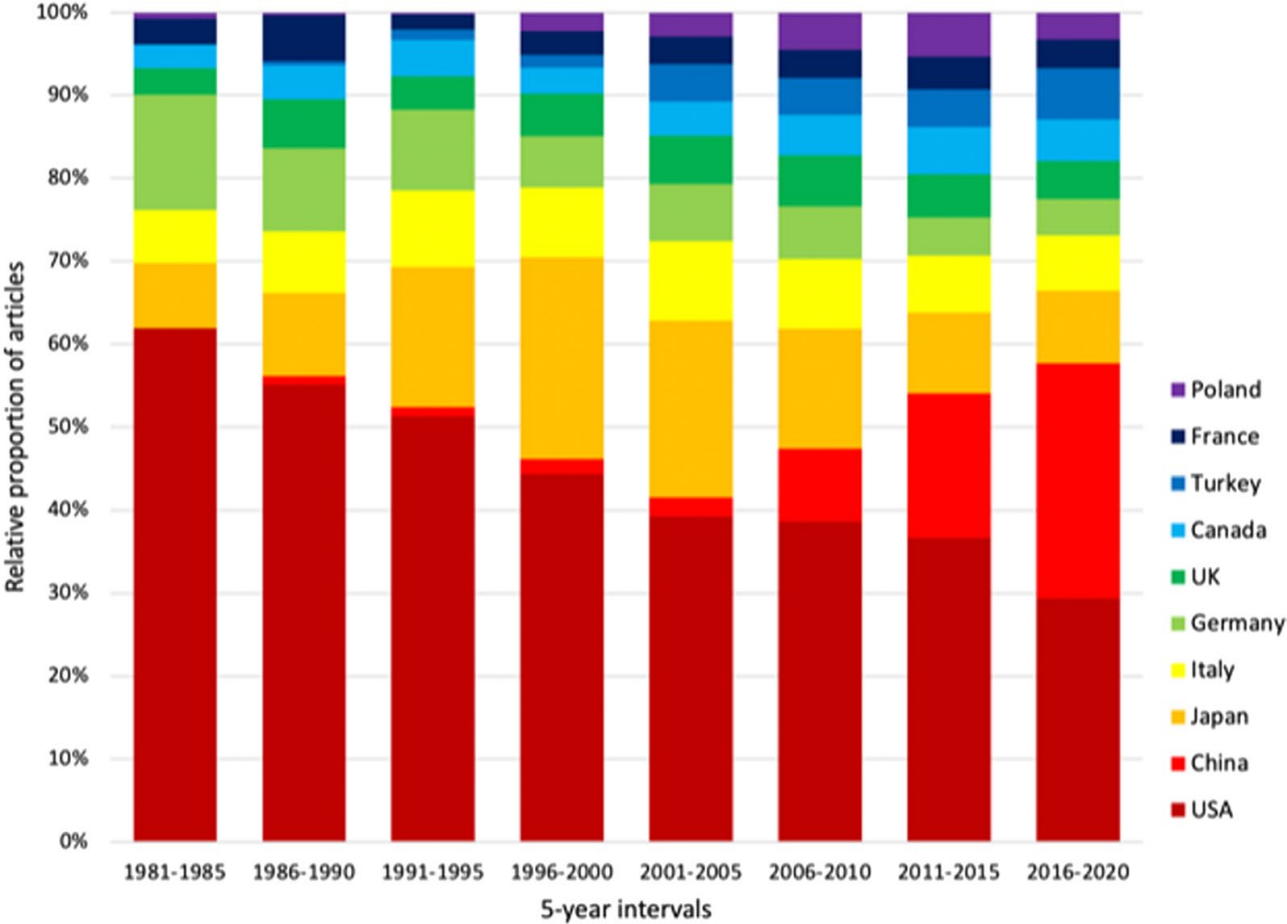
Relative proportions of number of the ten most productive countries in five year intervals from 1981 to 2020

From 1981 until 2020, we also stratified the scientific output of the 10 most productive countries in the field by 5 year-intervals and analyzed the proportional change of every county in the scientific output over time (Fig. 3). Here it was clearly visible, that the USA published over 60% of endometrial cancer articles in the field in 1981 until 1985. This percentage decreased to less than 30% in 2016 until 2020. In contrast, the relative publication output by China grew from 2% to almost 30% of the total publication output of the ten most active nations. For Japan, we could document an increase of the respective proportion in total articles published in endometrial cancer until 2000, which was followed by a visible decrease until 2020. The output of authors affiliated with Italy, the UK and France remained relatively stable whereas Poland and Turkey grew their scientific contributions to the field to a maximum percentage in 2016–2020.

**Fig. 4:**
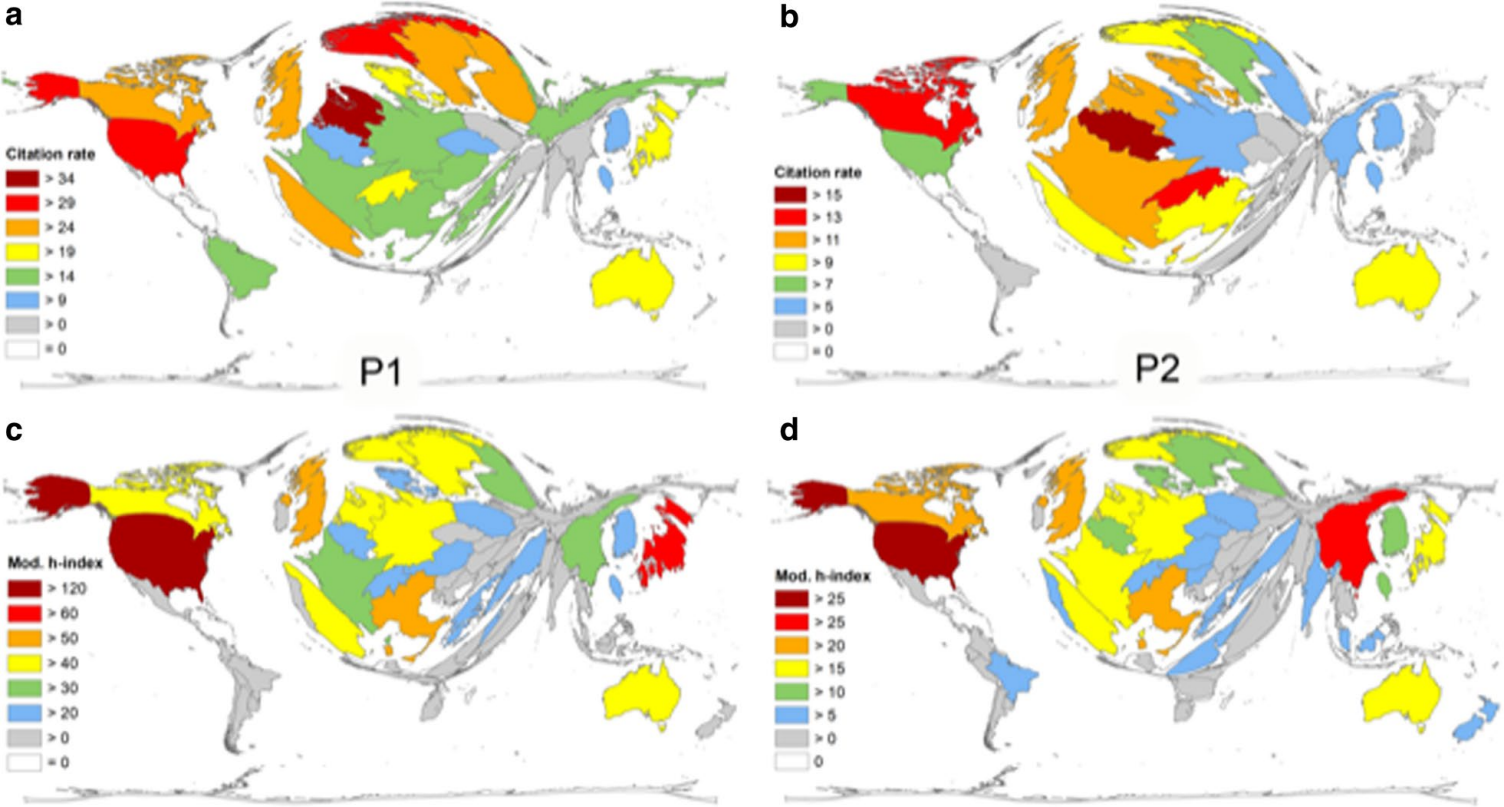
Density equalizing map projection of endometrial cancer citation parameters. Citation rate per country (threshold = 30 articles) in the period P1 = from 1900–2015 (**a**) and period P2 = period from 2016–2020 (**b**). Modified (country-specific) h-Index per country in the period P1 from 1900–2015 (**c**) and period P2 = period from 2016–2020 (**d**)

The average citation rates were calculated for countries with more than 29 articles. For P1, this analysis showed a leading position of the Netherlands with an average citation rate of 34.3 average citations per endometrial cancer publication (cR = average citations per endometrial cancer article). It was followed by the USA (cR = 32.5), Norway (cR = 31.4), Canada (cR = 28), Finland (cR = 25.8), Spain (cR = 24.9), the UK (cR = 24.5) and Sweden (cR = 24). In contrast to these Northern American and Western European countries, Asian countries had a lower citation rate with Japan (cR = 19.9), Taiwan (cR = 11.4), India (cR = 5.5) and China (cR = 9). In South America, only Brazil published more than 29 articles on endometrial cancer and was included in the analysis; each Brazilian article was cited 14.4 times (Fig. 4a). From 2016 until 2020, the pattern of leading countries in the field changed. Each article published by Belgian authors was cited around 16 times (cR = 15.9). In second to fifth positions, Switzerland, Canada, Netherlands and UK followed with cRs of 14.53, 14.50, 12.3 and 12.2 respectively. The USA and China were found ranked 12th (cR = 8.1) and 18th (cR = 5.1, Fig. 4b).

Finally, a country-specific Hirsch- index was calculated for all endometrial carcinoma publications. Out of the 79 countries with publications related to endometrial cancer, 63.4% (50 countries) had a modified H-index (HI) between 0 and 10. 25 countries had a HI between 11 and 50 and only 4 countries topped a HI of over 50 (Fig. 4c). The USA dominated the country-specific H-index analysis. Authors with US-American affiliations published 127 articles, which were cited at least 127 times (HI = 127). They were followed by Japan (HI = 65), the UK (HI = 51), Italy (HI = 51) and Canada (HI = 50). The top five countries were followed by European countries: Sweden (HI = 46), the Netherlands (HI = 46), Germany (HI = 45), Norway (HI = 43) and Spain (HI = 43). In South America, only Brazil had a H-index above ten (HI = 12). The leading African countries were Egypt and South Africa with a H-index of HI = 6. Again, the DEMP global map showed a distorted focus towards Northern America, Japan, and Western Europe. For P2, we could define the following ranking: USA (HI = 36), China (HI = 27), Canada (HI = 25), the UK (HI = 23), and Italy (HI = 22, Fig. 4d).

### Socio-economic analysis of endometrial cancer research

After the calculation of the above listed general parameters, also a country-specific benchmarking was performed with regard to socio-economic figures.

**Fig. 5:**
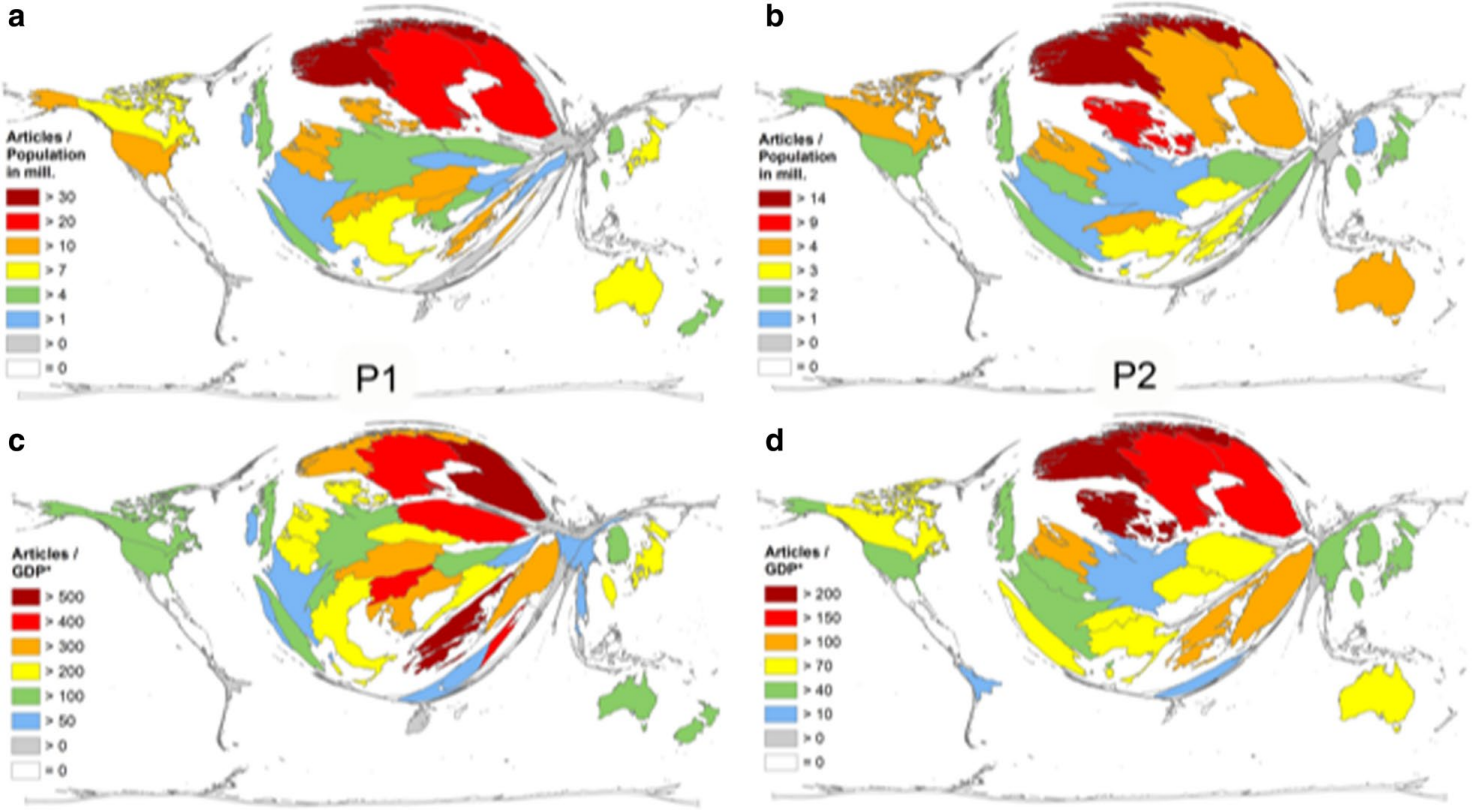
Density equalizing map projection of the ratio (Q1) of the number of endometrial cancer publications and population of the publishing countries in million inhabitants (World Factbook), classification of socio-economic status (World Bank) in the period P1 from 1900–2015 (**a**) and period P2 = period from 2016–2020 (**b**). Density equalizing map projection of the ratio (Q2) of the number of endometrial cancer publications and GDP in 1000 billion US-Dollar of the publishing countries (World Factbook), classification of socio-economic status (World Bank) in the period P1 from 1900–2015 (**c**) and period P2 = period from 2016–2020 (**d**)

As first quotient, the correlation between the country-specific amount of endometrial cancer specific publications as a measure for scientific activity in this field and the **number of citizens** was assessed (Q1 = article number (n) / citizens number in million). For P1, this analysis showed a marked difference between Scandinavian countries and the rest: Norway (Q1 = 35.80), Sweden (Q1 = 29.01) and Finland (Q1 = 27.19) had much higher values than i.e. the USA (Q1 = 10.01), or Japan (Q1 = 8.45, Fig. 5a). This pattern with Scandinavian countries in a leading position did not change for the time period from 2016 until 2020. Authors affiliated with Norway (Q1 = 14.81), Denmark, (Q1 = 9.65), Sweden (Q1 = 8.20), Finland (Q1 = 7.46) published very fruitfully in relation to the number of citizens. China was found on position 23 (Q1 = 9.65) and the USA occupied rank 13 (Q1 = 0.67, Fig. 5b).

As a next step, the research activity was related to the **gross domestic product** as proxy measure for the economic strength to invest in research (GDP, Fig. 5c). For this assessment the quotient Q2 was calculated by dividing the total number of endometrial carcinoma articles to the GDP in billion US-$. For 1900 until 2015, Greece was ranked first position with a Q2 = 579.83 calculated endometrial carcinoma related articles per 1000 billion US-$ GDP (Fig. 5). Finland was found in second place (Q2 = 527.29), followed by Sweden (Q2 = 494.65), and Israel (Q2 = 493.75). For Norway we calculated a Q2 of 367.85 endometrial carcinoma articles per GDP in billion US-$ (position 7). The USA published 183.18 endometrial carcinoma related articles per billion US-$ GDP and fell back at position 20. For P2, the picture did not change significantly. The four Scandinavian countries lead the ranking with Norway (Q2 = 213.87) followed by Denmark (Q2 = 203.93), Finland (Q2 = 171.40) and Sweden (Q2 = 162.62). Greece dropped to position 5 (Q2 = 134.25) and the USA was found in rank 19 (Q2 = 51.13, Fig. 5d).

**Fig. 6:**
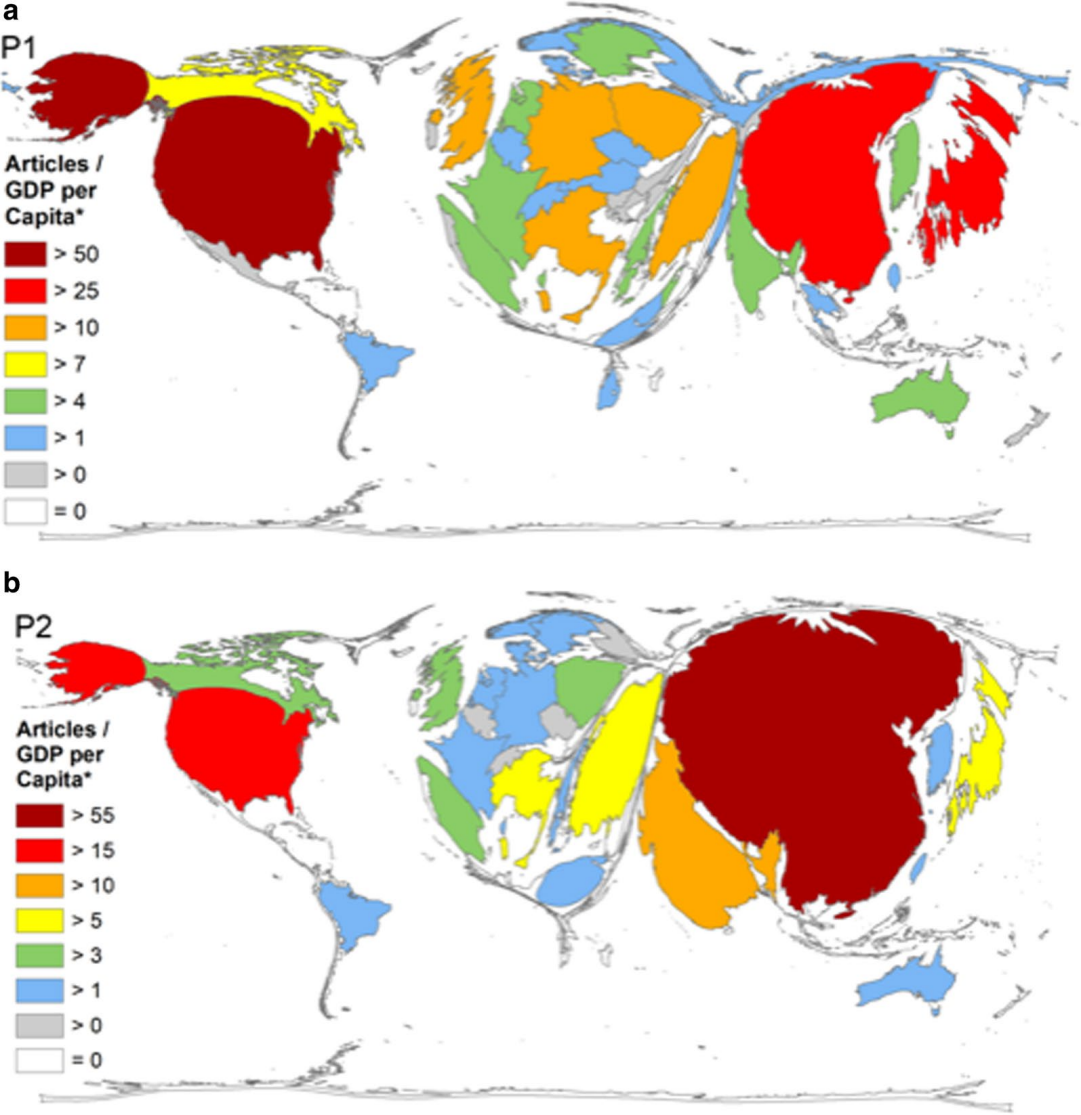
Density equalizing map projection of the ratio (Q3) of the number of endometrial cancer publications and GDP per Capita in billion US-Dollar of the publishing countries (World Factbook), classification of socio-economic status (World Bank) in the period P1 from 1900–2015 (**a**) and period P2 = period from 2016–2020 (**b**)

As third parameter (Q3, Fig. 6), the research activity measured in the number of articles was related to the **gross domestic product (GDP) per capita**. In P1, the USA took a lead position with a Q3 of 58.23 articles per billion US-$ per capita GDP (Fig. 6a). The Scandinavian countries Sweden (Q3 = 6.31), Finland (Q3 = 3.53) and Norway (Q3 = 2,79) fell back on ranks 13, 19 and 21. For P2, nations with large citizen counts were leading the field so China (Q3 = 59.42), the USA (Q3 = 16.56) and India (Q3 = 12.53) were located in rank 1 to 3. The pattern with the Scandinavian nations in lower ranked positions was visible in P2 (Sweden in rank 18, (Q3 = 1.63); Denmark in rank 21, (Q3 = 1.16); Norway in rank 22, (Q3 = 1.13); Finland in rank 24 (Q3 = 0.98, (Fig. 6b).

**Fig. 7:**
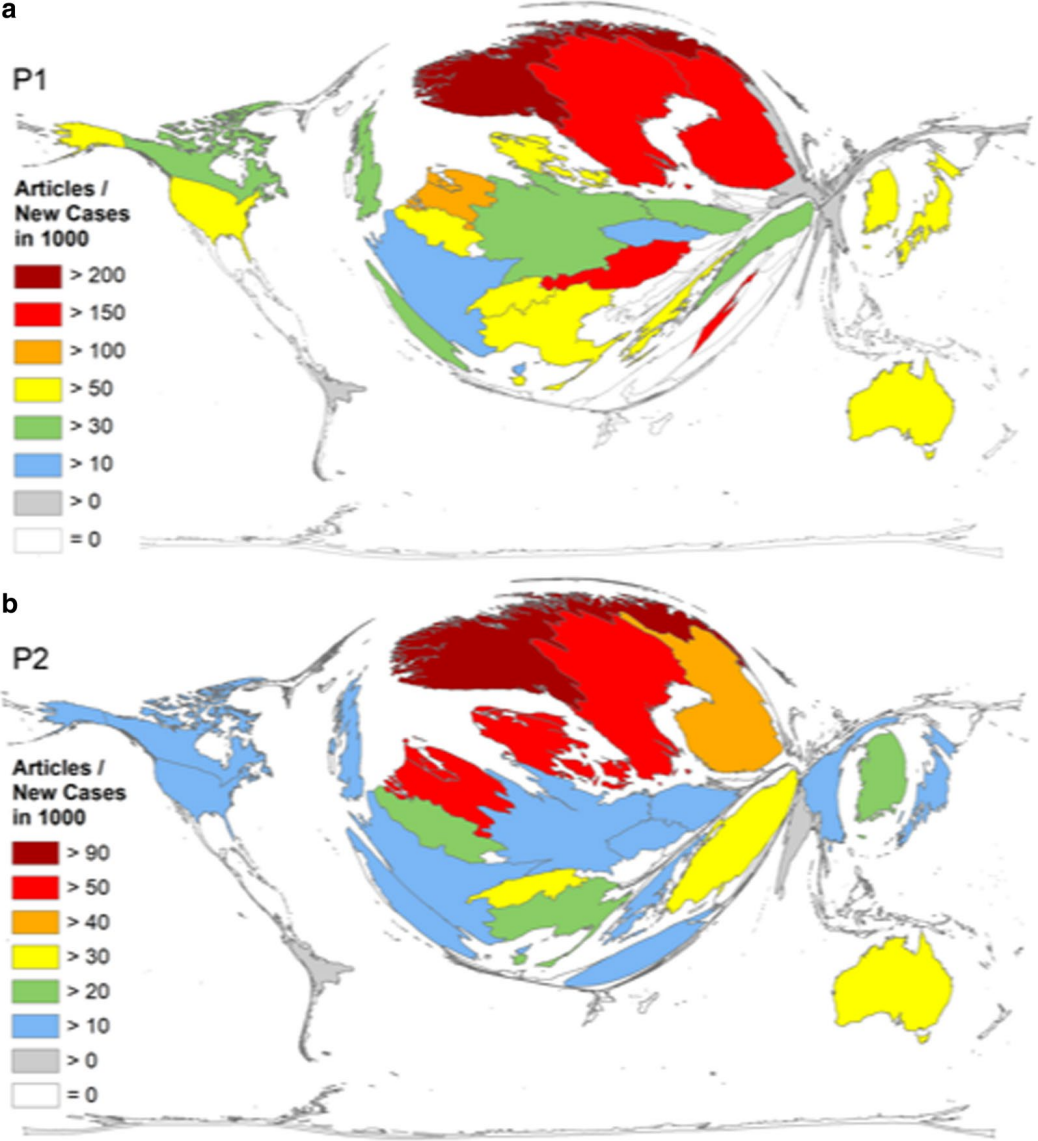
Density equalizing map projection of the ratio of the number of endometrial cancer publications per number of annual new cases of endometrial cancer in each country in the period P1 from 1900–2015 (**a**) and period P2 = period from 2016–2020 (**b**). Threshold of at least 30 endometrial cancer articles

A further disease-specific measure is the calculation of research activity in relation to the number of new endometrial cancer cases per year (Fig. 7a, Table 1). When published articles were related to the number of new cases per year in thousands, three Scandinavian countries exhibited the highest research activities in P1. Norway was first with a calculated 203.87 articles per 1000 yearly new endometrial cancer cases (Rinc), followed by Sweden (Rinc = 189.26) and Finland (Rinc = 163.8). The first non-Western European country was Australia positioned at rank 9 with a score of 72.37 endometrial cancer-specific articles per 1000 new cases. Countries that dominated the overall activities such as Japan or the USA had only values of 63.94 and 55.98 endometrial cancer-specific article per 1000 new cases, respectively. When we analyzed the time period from 2016 until 2020, three Scandinavian countries were again leading the field: Norway (Rinc = 97.87), Denmark (Rinc = 60.95) and Sweden (Rinc = 54.36) were positioned first, second and third, indicating that affiliated authors were publishing an effective amount of research in relation to the disease burden of endometrial cancer in the population. The USA and China, two countries with high incidences, were found in position 16 (Rinc = 16.65) and 22 (Rinc = 12.49), respectively (Fig. 7b).

**Table 1:** Endometrial cancer research activity (1900–2015) in relation to estimated annual new cases in single countries

Country	Annual new cases	Annual new cases/1000	Articles	Articles/Annual new cases in 1000
Norway	797	0.797	184	230.87
Sweden	1490	1.49	282	189.26
Finland	873	0.873	143	163.80
Austria	987	0.987	151	152.99
The Netherlands	2094	2.094	245	117.00
Denmark	886	0.886	85	95.94
Switzerland	1198	1.198	103	85.98
Belgium	1380	1.38	110	79.71
Australia	3040	3.04	220	72.37
Italy	8947	8.947	599	66.95
South Korea	3233	3.233	214	66.19
Japan	16,797	16.797	1074	63.94
Greece	2262	2.262	138	61.01
USA	57,004	57.004	3191	55.98
Iceland	36	0.036	2	55.56
Slovenia	419	0.419	22	52.51
Germany	10,429	10.429	519	49.77
Turkey	5463	5.463	253	46.31
Canada	8180	8.18	348	42.54
UK	10,677	10.677	409	38.31
Poland	7847	7.847	258	32.88
Spain	6784	6.784	215	31.69
Ireland	721	0.721	19	26.35
France	10,578	10.578	257	24.30
Croatia	900	0.9	20	22.22
Portugal	1069	1.069	22	20.58
Czech Republic	2144	2.144	42	19.59
Iran	1370	1.37	20	14.60
South Africa	1284	1.284	13	10.12
Hungary	1919	1.919	19	9.90
Egypt	1946	1.946	17	8.74
Thailand	2671	2.671	23	8.61
Slovakia	1048	1.048	9	8.59
China	73,253	73.253	611	8.34
Serbia	1594	1.594	11	6.90
Bulgaria	1320	1.32	9	6.82
Brazil	9105	9.105	47	5.16
Romania	2470	2.47	12	4.86
Argentina	2412	2.412	9	3.73
Russia	25,863	25.863	87	3.36
India	13,328	13.328	37	2.78
Pakistan	2881	2.881	7	2.43
Colombia	1583	1.583	3	1.90
Mexico	7266	7.266	13	1.79
Belarus	2239	2.239	4	1.79
Nigeria	1331	1.331	2	1.50
Ukraine	9328	9.328	9	0.96
Philippines	4048	4.048	2	0.49
Indonesia	6745	6.745	3	0.44

Epidemiologic data from GLOBOCAN 2018 [20]

### International endometrial cancer collaborations

**Fig. 8:**
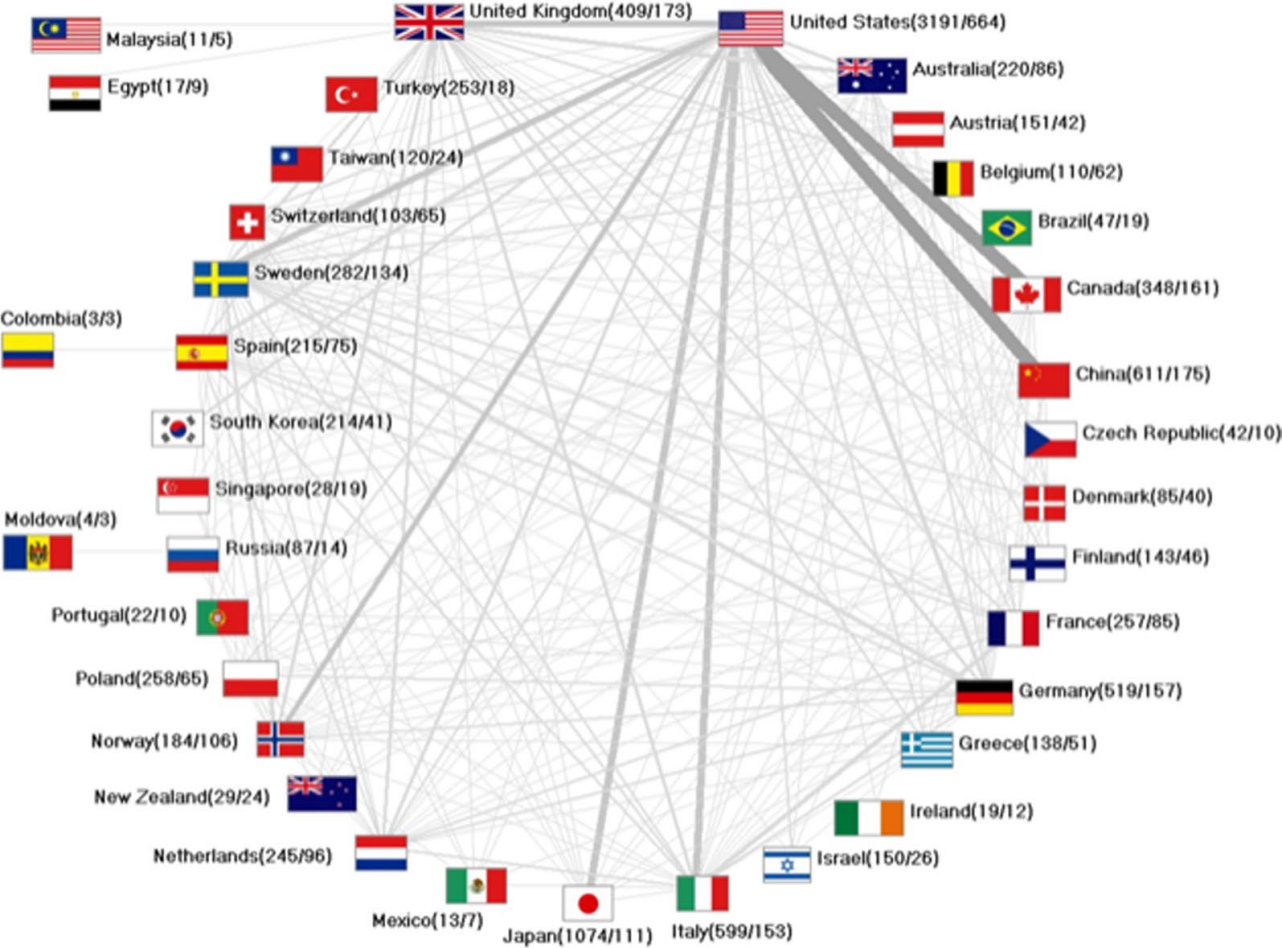
International network on endometrial cancer research

1120 articles were published by authors in a collaborative effort. 78.6% of these articles were issued by authors working in two different nations and joining forces (880 bilateral collaborations, n). 124 articles originated from trilateral collaborations. 45 items were based on collaborations between 4, and 21 articles between five countries. Among the collaborating countries, the USA was the most productive by far and was involved in 664 collaborations, followed by China (n = 175). Figure 8 illustrates the international endometrial cancer collaboration network with the USA an accentuated position.

### Endometrial cancer subject area analysis

**Fig. 9:**
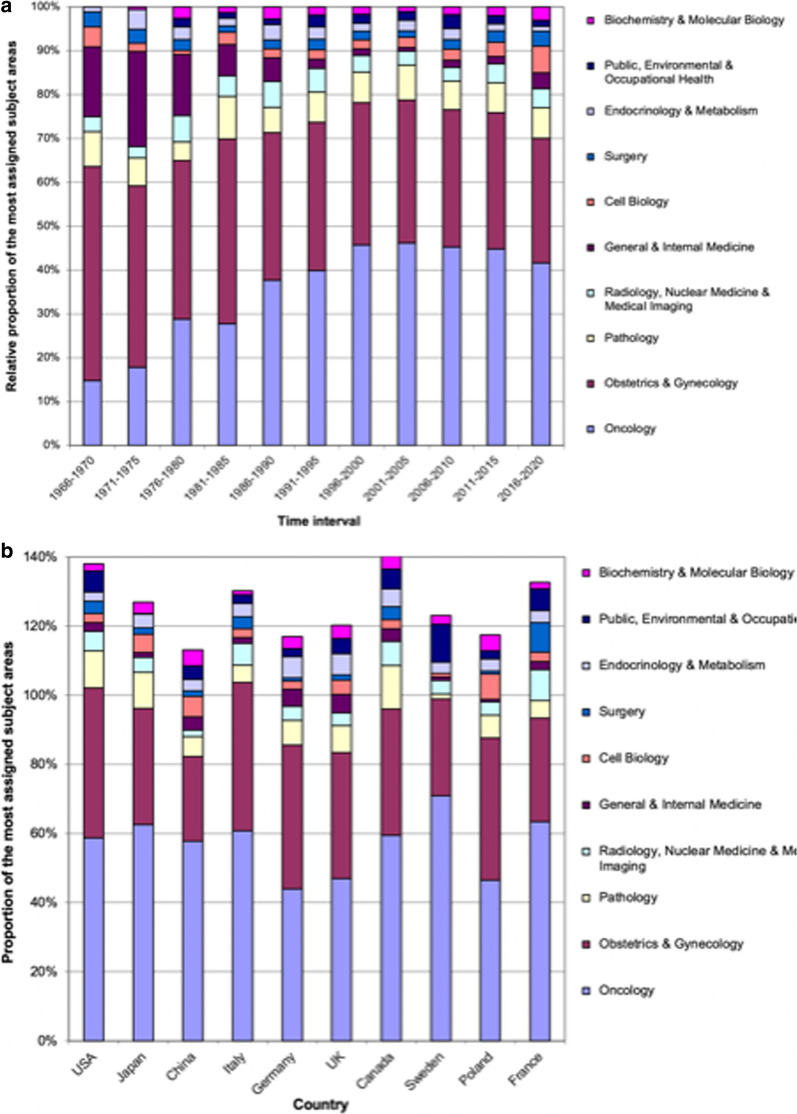
Subject areas of endometrial cancer research. **a** Relative proportion of the most assigned subject areas in 5-year intervals. **b** Proportion of the most assigned subject areas of the most publishing countries

The leading subject categories of endometrial cancer research were *Oncology* with 4,987 publications (n) cited 111,666 times (c), *Obstetrics & Gynecology* (n = 3,943, c = 72,334), and—following with a considerable gap—*Pathology* (n = 812 and c = 18,169) and *Radiology, Nuclear Medicine & Medical Imaging* (n = 481). When the relative proportion of the different subject areas was analyzed, it becomes evident that publication numbers in the area of *Public Health* decreased since 1966, and this particular specific subject area represented a very minor proportion of the last decade’s scientific output on endometrial cancer (Fig. 9a). We also conducted a subject area analysis for the ten most active countries in endometrial cancer research to identify their particular scientific focus: All of these countries were dominated by *Oncology* and *Obstetrics & Gynecology* and only minor differences appeared in other fields (Fig. 9b).

## Discussion

In order to present a global overview of research activities in an area of gynecological oncology since 1900, we here assessed the publication output on endometrial cancer by the use of the established NewQIS platform. During 115 years, a total of 9.141 original articles were identified. Our assessment of the number of published articles over time showed a continuous high research interest in this respective topic: From the 1970s onwards, the related publication rate increased continuously and exceeded 100 articles in 1982. This finding reflects groundbreaking discoveries in the field such as identification of estrogen and tamoxifen treatments as a significant risk factors and the definition of cancer subtypes. There is also a progressive rise noticeable from 2004 onwards, when annual publications more than doubled.

The USA was the most productive country among nations working on endometrial cancer. US-American publications gained the highest country-specific citation numbers and HI. Conversely, the article count in relation to the economic strength of the country dropped to rank 20, which indicates a rather less effective translation of large economic resources into scientific output compared to other countries. The outstanding position of two Asian nations—Japan and China—was remarkable. Both countries published many collaborative articles together with US-American scientists indicating fruitful collaborations between all three countries.

The interests of Japanese and Chinese scientists in endometrial cancer may be explained by the increasing incidences of this malignancy in both countries [22, 23]. For Japan is noteworthy that the numbers of affected women under 40 years of age are growing steadily [22], which suggests a local need to scientifically focus on fertility preserving strategies. For Chinese women, endometrial cancer is the second most common cancer of the female genital system [23]. Further, the 5-year relative survival rate is remarkably lower compared to Western countries [e.g. 55.1% in China [24]] versus 85% in the USA, https://www.cancer.org/content/dam/cancer-org/research/cancer-facts-and-statistics/annual-cancer-facts-and-figures/2020/cancer-facts-and-figures-2020.pdf) which might affect related research strategies and policies.

When the findings of our study were compared to existing data on research activity in other fields of gynecology similarly-structured assessments of research productivity focusing upon ovarian cancer [25], uterine myoma/fibroid [17] and breast cancer [12] might be of special interest: The analysis of Glynn et al. on breast cancer was limited to 63 years (1945–2008) [12]. In this time, a far higher amount was published on breast cancer (180,126 items). However, the absolute numbers of items is difficult to compare between both respective studies since Glynn et al. did not focus solely on research articles but also included other publications such as news, and reviews [12]. Still, the relative increase in research activity appears to be quite similar with the exception of more pronounced yearly spikes in the evolution of endometrial cancer research activities.

For the analysis of global scientific productivity on ovarian cancer we followed a structurally similar approach. Hence, both studies are better comparable. From 1900 to 2014, 23,378 articles on ovarian cancer were identified in comparison to the 9,141 articles on endometrial cancer. As in the present study, the USA displayed the highest research activity (n = 9,312 ovarian cancer-specific articles). However, Japan (rank 5) was not as productive in ovarian cancer compared to endometrial cancer research. In the socioeconomic benchmarking, Denmark was positioned first (1293.2 ovarian cancer-specific articles per 1000 billion US-$ GDP) and followed by Israel concerning the relation of ovarian cancer research activity to the total economic power index GDP. For endometrial cancer, Greece was the leading nation (579.83 articles per 1000 billion US-$ GDP) followed by Finland, Sweden and Israel. Our present data show that the relative research output in relation to the economic strength (as a proxy for a nation’s potential to invest in research) is higher for ovarian carcinoma than for endometrial cancer research. This finding might indicate a more pronounced global research interest  in ovarian cancer, which might be due to the higher disease burden of ovarian malignancies (e.g. high mortality and recurrence rates) and therefore the need for novel treatment strategies in this field.

For the global research output on uterine fibroids,  a similar pattern was shown in the socioeconomic analysis [17]. Here, Taiwan was positioned first (279.46 fibroid-related publications per 1000 billion USD GDP). It was followed by Israel and Finland. Interestingly, Israel and Finland were also identified to be highly active for endometrial and ovarian cancer research. Together, these findings might indicate that Scandinavian countries appear to be more active in female tumor research in terms of their socioeconomic capabilities than other Western countries. Aligning with these findings, the three Scandinavian countries Norway, Sweden and Finland published the most articles on endometrial cancer related to the local incidence of this malignancy in the respective countries. Thus, our findings may indicate that local research policy in Scandinavia is very successful and effective resulting in a high scientific productivity when related to investments in research and the local burden of the condition.

Most endometrial cancer articles were attributed to the subject area of “Oncology” and to “Gynecology and Obstetrics”, which was not surprising. Endometrial cancer has increasing prevalence rates in industrialized countries. Thus, prevention (e.g. in the area of patient awareness or lifestyle interventions) is key to decrease global numbers,  and research in the area of “Public Health” would be crucial for the alleviation of disease burden. Also, needs to improve research infrastructure and foster international collaborations with low-resource countries are further shortcomings we identified in our study that should be addressed in future efforts.

For limitations, we acknowledge an underestimation of the total article number related to endometrial cancer since we based our analysis only on  articles published in the Web of Science database. This source considers mainly English articles. For example, items published before the World Wars might not be taken fully into account since they were mostly written in German, which was the language of science used at this time. Furthermore, we regard our analysis of scientific quality as biased. Citations and the HI are proxy measures to gauge the recognition of articles in the scientific community rather than allowing for quantification of true quality. In this context, self-citations or the Matthew effect (the work of well known authors will be cited more compared to the publications of less known scientists) might skew the citation parameters. A further limitation is related to the search procedure: Only the title search tool was used. This was done in order to exclude false positive results and to guarantee the representativity of the identified articles concerning endometrial cancer.

A third limitation is the use of a single database. This limitation is due to the fact, that the second available system of the study groups platform, the PubMed, does not provide the possibility for a citation analysis.

Furthermore, the influence of publication bias needs to be discussed. Publication bias occurs when the outcome of a study influences the decision whether to publish it or not. However, there are no quantitative data available pointing to country-specific issues in publication biases.

Although NewQIS studies usually do not focus upon the research activities of single authors but we here want to make an exception to honor a scientist who died far too soon: *Dr. Helga Birgitte Salvesen* (23 December 1963 to 20 January 2016) was professor of gynecology and obstetrics, at the Haukeland University Hospital Bergen in Norway. In the present set articles specifically related to endometrial cancer, she was as a scientist in the leading positions in nearly every category: She was an author of 89 articles of the presently analyzed 9,141 articles. These articles were cited 3,322, 26 items were at least cited 26 times (modified, endometrial carcinoma-specific H-index of 26). Due to her research, her country Norway was top ranked in the assessment of endometrial cancer articles in relation to newly diagnosed cases with 230,87 calculated articles per 1000 new endometrial cancer cases. Out of her numerous important contributions, i.e. studies on the prognostic significance of angiogenesis and Ki-67, p53, and p21 expression in a population-based endometrial carcinoma study [26], many were cited more than a hundred times. A study performed within the Cancer Genome Atlas Research Network on the integrated genomic characterization of endometrial carcinoma received more than 1000 citations [27]. The scientific community working on endometrial cancer will surely miss her trailblazing contributions.

## Conclusions

The present NewQIS study on endometrial cancer reveals that this female cancer receives a global research attention, which steadily increases. But it is evident that the magnitude of research activities appears to be less than that of ovarian cancer research. As with other fields of oncology, the global landscape is dominated by the USA. In contrast to other fields, two Asian countries—Japan listed in position 2 and China in position 3—are among the top three active countries. A strong dedication of single countries to endometrial cancer research can be visualized when research productivity is related to socio-economic features. Here, Scandinavian countries, Greece and Israel appear to be very strong. Our study also illustrates the lack of research activities among many countries with a high burden of diseases. This should be a target for future public and global health strategies since these countries need to be empowered to be present in collaborative research networks leading to mutual benefits due to the exchange of data.

## Data Availability

The datasets used and/or analyzed during the current study available from the corresponding author on reasonable request.

## Abbreviations

CR: Citation rate
DEMP: Density equalizing map projections
FIGO: Fédération Internationale de Gynécologie et d’ Obstétrique
GDP: Gross domestic product
HI: Hirsch index
IARC: International Agency for Research on Cancer
NewQIS: New Quality and Quantity Indices in Science
WHO: World Health Organization
WoS: Web of Science
